# Comparative genomic and transcriptome analysis of *Bacillus velezensis* CL-4 fermented corn germ meal

**DOI:** 10.1186/s13568-023-01510-5

**Published:** 2023-01-23

**Authors:** Long Chen, Zihui Qu, Wei Yu, Lin Zheng, Haixin Qiao, Dan Wang, Bingdong Wei, Zijian Zhao

**Affiliations:** 1 Institute of Animal Nutrition and Feed, Jilin Academy of Agricultural Sciences, No. 186 Dong Xinghua Street, Gongzhuling, 136100 Jilin China; 2 Institute of Agro-Food Technology, Jilin Academy of Agricultural Sciences, No. 1366 Cai Yu Street, Changchun, 130033 Jilin Province China; 3 Information Application Department, Jilin Intellectual Property Protection Center, Changchun, 130000 China

**Keywords:** *B. velezensis* CL-4, Comparative genomics, Transcriptome, Lignocellulose-degrading enzymes, Corn germ meal

## Abstract

*Bacillus,* an excellent organic-degrading agent, can degrade lignocellulose. Notably, some *B. velezensis* strains encode lignocellulases. However, their ability to degrade lignocellulose in fermented feed is not much appreciated. This study performed a comparative genomic analysis of twenty-three *B. velezensis* strains to find common carbohydrate-active enzymes (CAZymes) encoding genes and evaluated their potential to degrade lignocellulose. The comparative genomic and CAZyme database-based analyses identified several potential CAZymes genes that degrade cellulose (GH1, GH4, GH5, GH13, GH16, GH32, PL1, and PL9), hemicellulose (GH11, GH26, GH43, GH51, and CE3) and lignin (AA4, AA6, AA7, and AA10). Furthermore, Illumina RNA-seq transcriptome analysis revealed the expression of more than 1794 genes in *B. velezensis* CL-4 fermented corn germ meal at 48 h (FCGM 48 h). Gene ontology analysis of expressed genes revealed their enrichment in hydrolase activity (breaking the glycosyl bonds during carbohydrate metabolism), indicating the upregulation of CAZymes. In total, 58 differentially upregulated CAZymes-encoding genes were identified in FCGM 48 h compared to FCGM 0 h. The upregulated CAZymes-encoding genes were related to cellulose (6-phospho-β-galactosidase and 6-phospho-α-glucosidase), starch (α-glucosidase and α-amylase), pectin (pectin lyase), and hemicellulose (arabinan endo-1,5-α-L-arabinosidase, xylan 1,4-beta-xylosidase, α-N-arabinofuranosidase, and acetyl xylan esterase). Importantly, arabinoxylan degradation mainly occurred in FCGM 48 h, followed by partial degradation of cellulose, pectin, and starch. This study can support the development of enzymatic cocktails for the solid-state fermented feed (SFF).

**Supplementary Information:**

The online version contains supplementary material available at 10.1186/s13568-023-01510-5.

## Key points

- Comparative genomics and transcriptomics revealed that *B. velezensis* encodes a series of lignocellulosic enzymes capable of degrading polysaccharides.
- Arabinoxylan degradation mainly occurs after 48 h of *B. velezensis *CL-4 fermentation, followed by the partial degradation of cellulose, pectin and starch.
- Exogenous cellulase could significantly improve the fermentation quality of corn germ meal.

**Supplementary Information:**

The online version contains supplementary material available at 10.1186/s13568-023-01510-5.

## Introduction

Soybean meal (SBM) is an important protein feed component in animal diets. With the rapid development of the breeding industry, increasing consumption of livestock products, and sharp fluctuation in SBM price, it has become urgent to reduce or find a replacement for SBM. Corn germ meal (CGM) is an unconventional protein feed resource with a high yield, wide source, and low price in the Jilin province of China, and the annual supply of corn germ meal is ~ 1 × 10^7^ tons. Its crude protein content is about 22.6%, while the fiber content is close to corn DDGS (Distillers Dried Grains with Solubles) (Zhang et al. 2019b). Also, cellulose and arabinoxylan are the main fiber components of CGM, the NDF (Neutral Detergent Fiber) content is about 50.44%, and the apparent total digestive tract digestibility of fiber components is less than 50% (Jaworski et al. 2015). Animal nutritionists have made some progress on CGM by optimizing its proportion in diets and adding enzyme preparations to replace part of corn and SBM in diets (Shi et al. 2019). However, the high fiber content of CGM is a major limiting factor in its application in animal diets, which reduces growth performance and feed conversion rate (Zhang et al. 2013). In our previous study, we applied sodium bicarbonate pretreatment and *B. velezensis* CL-4 fermentation to effectively improve the nutritional values of CGM. Notably, the degradation rates of cellulose and hemicellulose of CGM were 20.33% and 71.35%, respectively (Chen et al. 2022). In recent years, microbial fermentation has become an effective way to improve the utilization rate and nutritional quality of plant protein feed (Olukomaiya et al. 2019). One report compared the digestibility, metabolizable energy, and standardized ileal digestibility of amino acids in fermented corn germ meal (FCGM) and SBM, and evaluated the effect of FCGM replacing SBM in diets of growing pigs. They found that 11.8% of SBM can be replaced by FCGM to obtain the best daily gain in growing pigs (He et al. 2022). So far, only a few reports used microbial fermentation to improve the nutritional value of CGM and showed that FCGM could improve animal performance and replace SBM. The present research on *B. velezensis* mainly focuses on biological control and the promotion of plant growth (Adeniji et al. 2019). After the inclusion in the European Union Safety Qualification (QPS) List of recommended biologics in 2020, *B. velezensis* use in livestock and poultry has markedly increased (Khalid et al. 2021). Thus far, there are only a few reports about *B. velezensis* fermented feeds showing its potential advantages in lignocellulose degradation, especially in soya bean fermented meals (Chen et al. 2021).

This study performed comparative genomic analysis of twenty-three *B. velezensis* strains from different sources to search for common CAZymes and then performed transcriptomics analysis to identify the lignocellulase-encoding genes that were significantly upregulated during the *B. velezensis* CL-4 fermentation of pretreated CGM. Our results may help improve the co-fermentation technology in the fermented feed industry.

## Materials and methods

### Microorganisms and fermentation

*Bacillus velezensis* CL-4 strain (CTCC No: M2020811) was obtained from the cecal contents of broilers. CGM was purchased from Gongzhuling Wellhope Animal Husbandry Co., LTD. The culture and activation of *B. velezensis* CL-4 and the selection and pretreatment of CGM were performed as described previously (Chen et al. 2022). Before fermentation, CGM was pretreated with sodium bicarbonate to neutralize pH at about 7.0. The pretreated CGM was inoculated with *B. velezensis* CL-4 (6.0 log CFU/g) for fermentation at 37 °C for 48 h; the final moisture content was maintained at 50% by covering with a sterile membrane having breathing holes for air exchange. All samples were tested in quintuplicate. Control (0 h) and fermented (48 h) samples were subjected to Illumina RNA-seq.

### Mining of homologous genes related to lignocellulose degradation

The whole genome information of the twenty-three *B. velezensis* strains was retrieved from the GenBank DNA database. HMMER (version 3.0) software (Krogh et al. 1994) was used to predict the presence of CAZymes in the genome sequences. For CAZymes analysis, the protein sequences of the twenty-three reference genomes were downloaded from the GenBank database. All the protein sequences to be analyzed were combined into a file as a database, which was subjected to all-vs-all Blastp analysis; the series alignment threshold was set to 1e-10. The sequence alignment results were processed with the OrthoFinder (version 2.2.7) software (Emms and Kelly 2019). OrthoFinder algorithm was used to cluster gene families, and the “Inflation” adopted for clustering was set to 1.5. Finally, a self-made Perl script was used to sort out and count the clustering results.

### Total RNA extraction and illumina RNA-seq

The bacterial total RNA was extracted by using the Trizol reagent (Ambion, Texas, USA), following the manufacturer’s recommendations. The sample quality and integrity were analyzed by a NanoDrop spectrophotometer (Thermo Scientific, Massachusetts, USA) and Bioanalyzer 2100 system (Agilent, California, USA), respectively. rRNA was removed from total RNA using the Zymo-seq ribofree total RNA library kit (Zymo Research, California, USA) following the manufacturer's instructions. For hybridization, the 3ʹ ends of the DNA fragments were adenylated and ligated to Illumina PE adapter oligonucleotides. cDNA fragments of 400 to 500 bp in length were selected and purified using the AMPure XP system (Beckman Coulter, CA, USA). Illumina PCR primers cocktail was used to selectively enrich the DNA fragments of the two ligating adaptor molecules by a 15-cycle PCR reaction. The reaction was performed under the following conditions: 98 °C for 30 s, followed by 15 cycles of 98 °C for 10 s, 60 °C for 30 s, and 72 °C for 30 s, and end at 72 °C for 5 min. Agilent’s highly sensitive DNA analysis was performed to quantify samples using a Bioanalyzer 2100 system (Agilent, California, USA). The sequencing libraries were sequenced by Shanghai Personal Biotechnology Company on a NovaSeq 6000 platform (Illumina Novaseq, CA, USA).

### Bioinformatics analysis

#### Data quality control and read mapping

After calculating the quality information of raw data in FASTQ format, Cutadapt (v1.15) software (Kechin et al. 2017) was used to filter the original data. Next, we used Bowtie2 (v2.2.6) software (Langmead and Salzberg 2012) to map filtered reads to the *B. velezensis* CL-4 genome (Genbank accession CP081304.1).

#### Expression level and differential expression analysis of CAZymes

HTSeq (v0.9.1) (Anders et al. 2015) was used to measure the read count of the original expression level of the genes. We used the FPKM (fragments per kilobase of exon per million fragments mapped) to normalize the expressed genes to make the expression levels of different genes and samples comparable. The differential expressed mRNAs were identified by DESeq (v1.30.0) (Wang et al. 2010b) with the *P-*value and log_2_ fold change set to < 0.05 and > 1, respectively. The CAZymes-encoding genes were screened against the CAZymes database (http://www.cazy.org/).

#### Gene ontology enrichment analysis

We examined the number of differentially enriched genes for the respective GO (Gene Ontology) terms. The top GO terms and P values were calculated based on the hypergeometric distribution method (Midorikawa et al. 2018).The main biological functions of differentially expressed genes (DEGs) were determined based on significantly enriched GO terms.

#### Validation of RNA-seq-derived DEGs by RT-qPCR

We followed the MIQE guidelines (Bustin et al. 2009) for RT-qPCR validation of DEGs identified from transcriptome analysis. Primer Express R software (Applied Biosystems) and OligoAnalyzer 3.1-IDT were used to design and evaluate all specific primers. The total bacterial RNA was extracted by using the Trizol reagent (Ambion, Texas, USA), following the manufacturer’s recommendations and treated with 5U of Amplification Grade DNase I (Invitrogen, Carlsbad, CA, USA) for 10 min at 70 °C. After DNase I denaturation, the RNA sample was chilled on ice and immediately used with Oligo(dT) 20 primers (Invitrogen, Carlsbad, CA, USA) and SuperScript R II Reverse Transcriptase (Invitrogen, Carlsbad, CA, USA) to synthesize cDNA. For RT-qPCR analysis, the amplification was performed with a Step One Plus Real-Time PCR System (Applied Biosystems). The reaction was performed under the following conditions: 95 °C for 5 min, followed by 40 cycles of 95 °C for 15 s, 60 °C for 40 s. Amplicons were identified by melting curve analysis and the 16 s rRNA gene was employed as a stable reference gene after cycle completion. Three biological and three technical replicates were performed for each sample. Gene expression levels were estimated using the 2−^ΔΔCT^ method (Livak and Schmittgen 2001).

#### Statistical and bioinformatics analysis

Data were analyzed by SPSS 24.0 software (SAS Inc., Chicago, IL, USA). Differences between means values were determined using one-way analysis of variance (ANOVA) and Duncan's test. Data with *P* < 0.05 were considered significant.

## Results

### Comparative genomic analysis of CAZymes from the *B. velezensis* strains

**Fig. 1:**
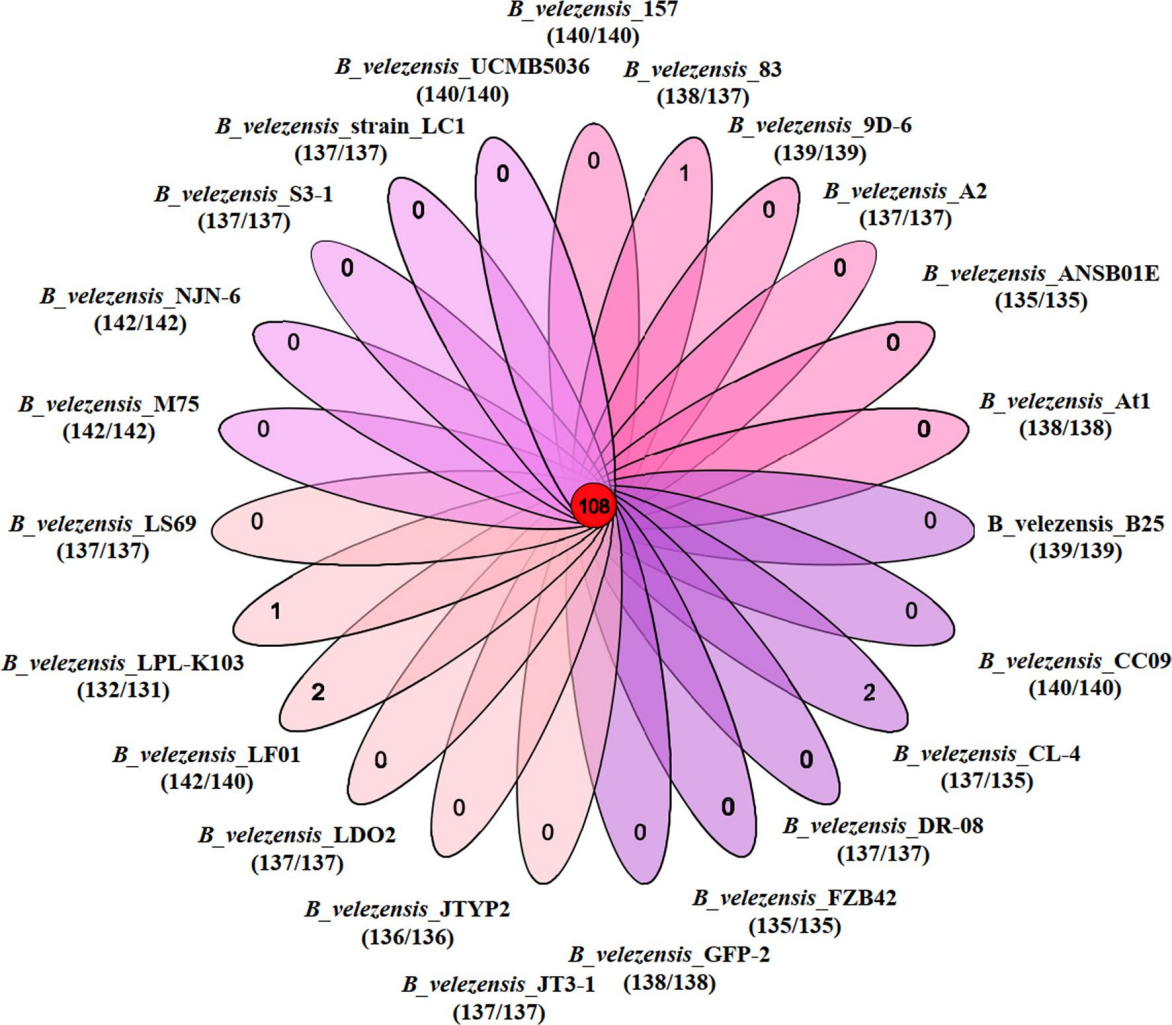
The total number of genes in each species and genes/gene family were obtained by comparative CAZyme analysis of the twenty-three *B. velezensis* strains. Side circles represent the total number of genes in a species, and the center circle represents the number of gene families shared by the *B. velezensis* strains. The total number of genes/gene families shared by a *B. velezensis* strain is shown in parenthesis

The assembled genome of *B. velezensis* CL-4 (PRJNA754316) was compared to the genomes of the other twenty-two *B. velezensis* strains (Fig. 1). The corresponding accession numbers and other details about the *B. velezensis* strains are listed in Additional file 1: Table S1. The glycosyl transferases (GT), auxiliary activities (AA) and polysaccharide lyases (PL) family numbers were the same in these strains, whereas the strain CL-4 contained more glycoside hydrolases (GH) and carbohydrate-binding modules (CBM) family members and less carbohydrate esterases (CE) members. Annotated common genes encoding lignocellulose-degrading enzymes from the twenty-three *B. velezensis* strains are shown in Table 1, which mainly involves the degradation of cellulose, starch and xylan.

**Table 1:** Annotated common genes encoding lignocellulose-degrading enzymes in the twenty-three *B. velezensis* strains

Classification	CAZy	Predicted function	EC number
Cellulose-related	GH1	6-phospho-β-galactosidase	EC 3.2.1.85
	GH1	6-phospho-β-glucosidase;	EC 3.2.1.86
	GH4	6-phospho-β-glucosidase	EC 3.2.1.86
	GH4	6-phospho-α-glucosidase	EC 3.2.1.122
	GH4	α-galactosidase	EC 3.2.1.22
	GH5	endo-1,4-β-glucanase	EC 3.2.1.4
	GH13	a-glucosidase	EC 3.2.1.20
	GH32	endo-levanase	EC 3.2.1.65
	GH13	a-amylase	EC 3.2.1.1
	GH16	β-1,3(4)-glucanase	EC 3.2.1.73
	PL1	pectin lyase	EC 4.2.2.10
	PL1	pectate lyase	EC 4.2.2.2
	PL9	pectate lyase	EC 4.2.2.2
Hemicellulose-related	GH11	endo-β-1,4-xylanase	EC 3.2.1.8
	GH26	endo-1,4-β-mannosidase	EC 3.2.1.78
	GH30	glucuronoxylanase	EC 3.2.1.–
	GH43	Arabinan endo-1,5-α-L-arabinosidase	EC 3.2.1.99
	GH43	arabinoxylan arabinofuranohydrolase	EC 3.2.1.–
	GH43	1,4-β-xylosidase	EC 3.2.1.37
	GH51	α-N-arabinofuranosidase	EC 3.2.1.55
	CE3	acetyl xylan esterase	EC 3.1.1.72
	CE3	acetyl xylan esterase	EC 3.1.1.72
Lignin-related	AA4	vanillyl-alcohol oxidase	EC 1.1.3.38
	AA6	1,4-benzoquinone reductases	EC 1.6.5.6
	AA7	FAD-binding protein	EC 1.1.3.–
	AA10	copper-dependent lytic polysaccharide monooxygenases (LPMOs)	EC 1.14.99.53

### Transcriptome of *B. velezensis* CL-4 during fermentation of CGM

#### Sequence metrics

Sequence quality statistics showed that the average length of the detected mRNA sequences was 380 bp, and the quality scores Q20 and Q30 were 99.0 and 99.9%, respectively. A large number of sequences with Q20 and Q30 > 90% are mentioned in Additional file 1: Table S2, indicating the high accuracy of sequencing. Also, the coverage of the obtained sequences on the genome of *B. velezensis* CL-4 was > 97% (Additional file 1: Table S3). Furthermore, principal component analysis of transcriptome reads revealed that the samples in respective groups were close to each other, indicating good repeatability/accuracy. Also, a marked separation between the reads of 0 and 48 h fermentation samples indicated significant differences in the transcriptome of the two samples (Additional file 1: Fig. S1). Pearson correlation coefficient of 0.8–1 indicated a high correlation between the expression patterns of the two samples (Additional file 1: Fig. S2). The SRA (Sequence read archive) accession PRJNA874839 were submitted to the NCBI SRA database.

#### Modulation of gene expression and CAZyme-encoding genes

Compared with the FCGM-0 h (control group) group, there were 1794 DEGs in the FCGM-48 h group, including 971 upregulated and 823 downregulated genes. Among them, 58 DEGs were CAZyme-encoding genes (all upregulated), including 13 DEGs encoding GH family proteins, 6 DEGs encoding CEs, 1 DEGs encoding AA proteins, 6 DEGs encoding CBMs (carbohydrate-binding modules), 1 DEGs encoding PLs and 31 DEGs encoding GTs. Among the 58 enzyme-encoding DEGs, most were involved in hemicellulose hydrolysis including arabinan endo-1,5-α-L-arabinosidase, xylan 1,4-beta-xylosidase, α-N-arabinofuranosidase, and acetyl xylan esterase. Genes related to cellulose and pectin depolymerization were also present (Table 2), such as those encoding 6-phospho-β-galactosidase, 6-phospho-α-glucosidase, α-glucosidase, α-amylase, copper-dependent lytic polysaccharide monooxygenases (LPMOs), and pectin lyase.

**Table 2:** CAZymes-encoding genes with significantly increased expression in the transcriptome of *B. velezensis* CL-4 fermented corn germ meal

NCBI gene code	EC	CAZy Family	Enzyme description	Log_2_Fold change
gene-K4L72_RS06255	3.2.1.85	GH1	6-phospho-β-galactosidase	1.48
gene-K4L72_RS04325	3.2.1.122	GH4	6-phospho-α-glucosidase	6.61
gene-K4L72_RS15355	3.2.1.20	GH13	α-glucosidase	2.13
gene-K4L72_RS01625	3.2.1.1	GH13	α-amylase	1.94
gene-K4L72_RS14170	3.2.1.99	GH43	Arabinan endo-1,5-α-L-arabinosidase	2.15
gene-K4L72_RS08970	3.2.1.37	GH43	xylan 1,4-beta-xylosidase	5.29
gene-K4L72_RS19300	3.2.1.99	GH43	Arabinan endo-1,5-α-L-arabinosidase	4.58
gene-K4L72_RS14125	3.2.1.55	GH51	α-N-arabinofuranosidase	2.03
gene-K4L72_RS14020	3.2.1.55	GH51	α-N-arabinofuranosidase	3.36
gene-K4L72_RS19275	4.2.2.10	PL1	Pectin lyase	1.18
gene-K4L72_RS13250	3.1.1.72	CE6	Acetyl xylan esterase	1.18
gene-K4L72_RS04250	3.1.1.72	CE4	Acetyl xylan esterase	2.19
gene-K4L72_RS08580	3.1.1.72	CE4	Acetyl xylan esterase	3.09
gene-K4L72_RS09050	1.14.99.–	AA10	Copper-dependent lytic polysaccharide monooxygenases (LPMOs)	1.26

#### Gene ontology enrichment analysis of DEGs

**Fig. 2:**
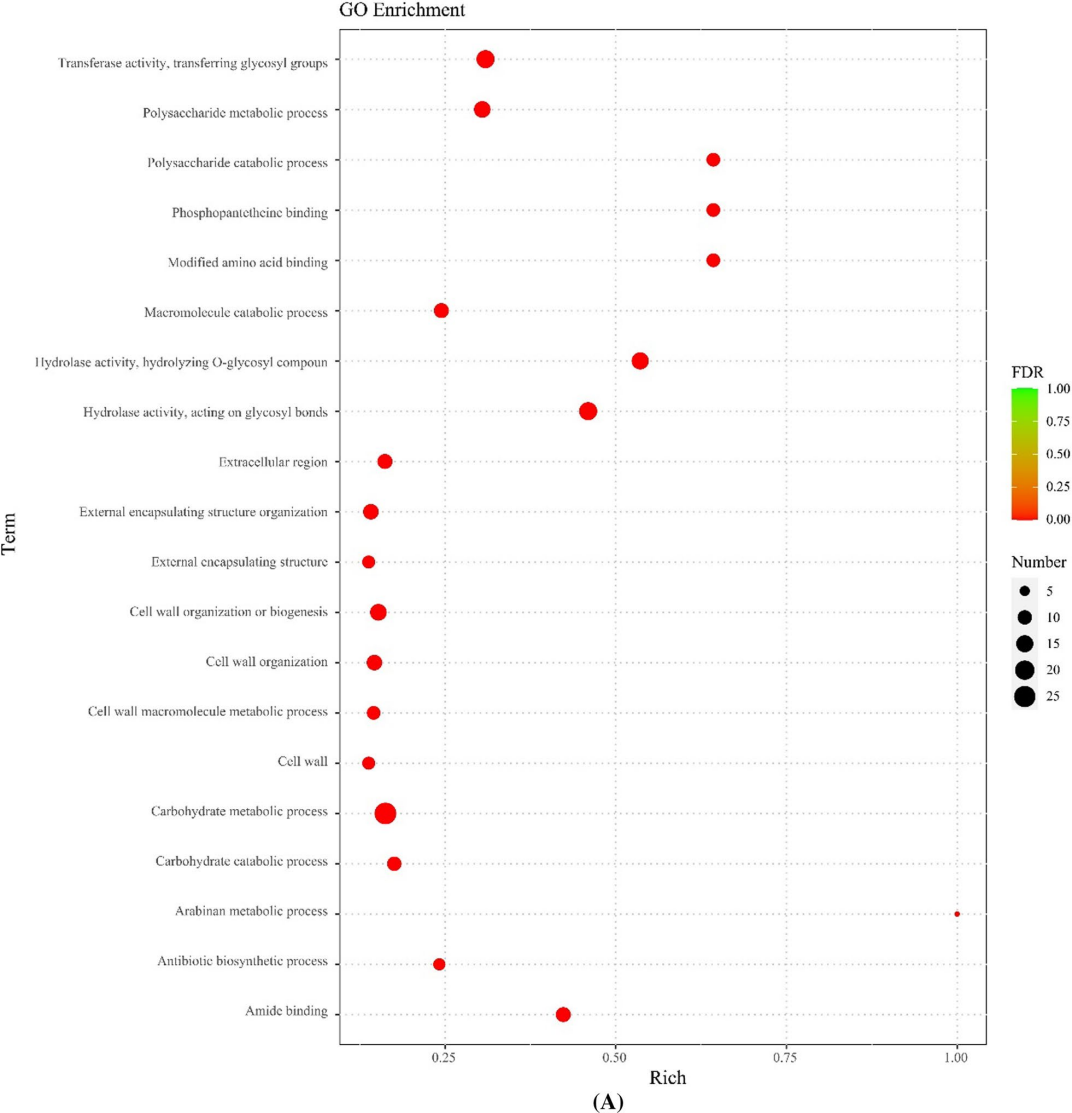

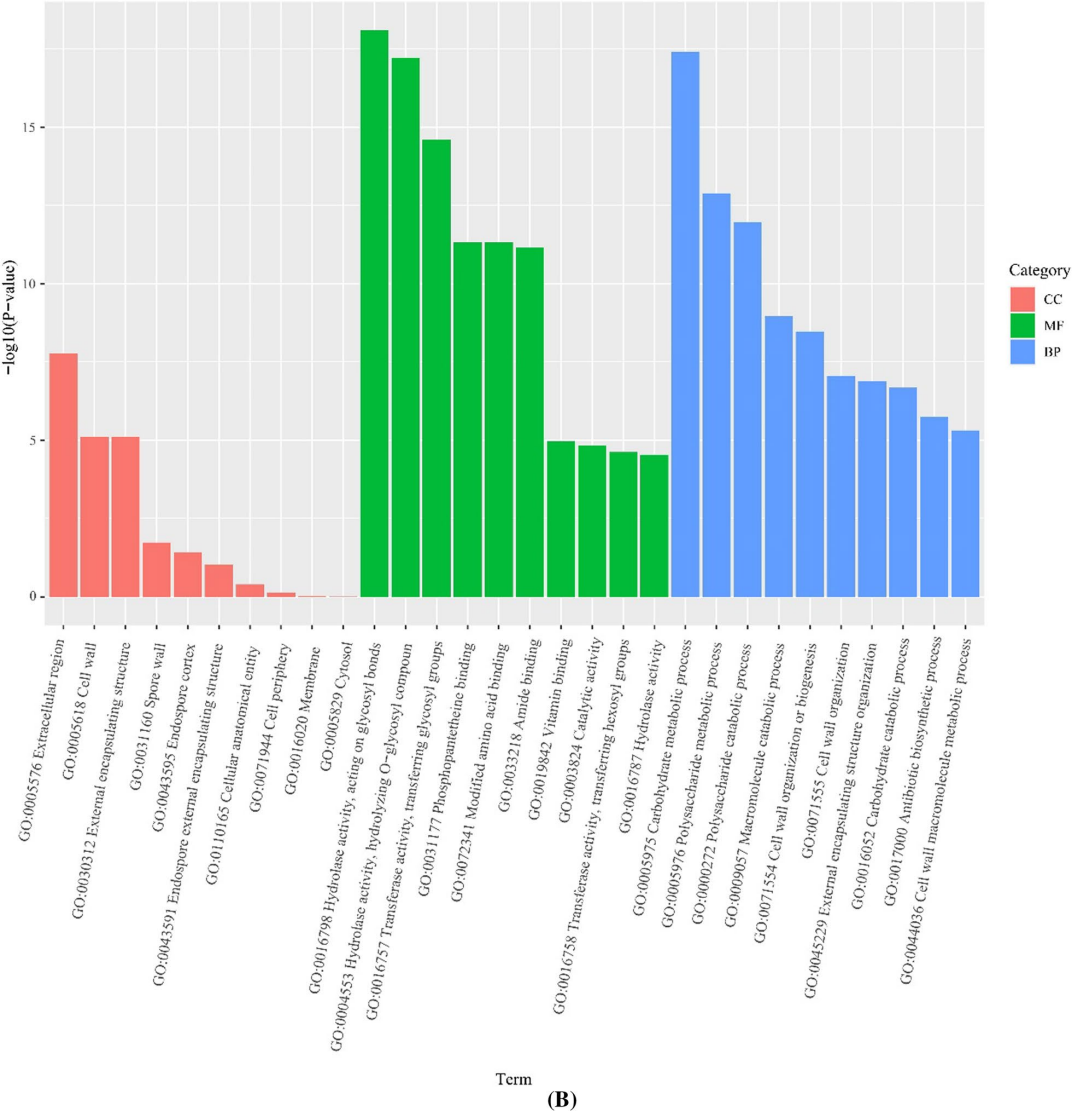
Gene ontology (GO) enrichment analysis of differentially expressed genes in *B. velezensis* CL-4 between the FCGM-0 h and FCGM-48 h groups. **A** A bubble diagram and **B** bar chart show the results of the GO enrichment analysis. MF stands for molecular function, BP for biological processes, and CC for cellular components

The DEGs were subjected to GO enrichment classification to find enrichment for molecular functions (MF), biological processes (BP), and cell components (CC). The top 10 GO terms with the minimum *P*-value (most significant enrichment) for respective GO classification are shown in Fig. 2A. Compared with FCGM-0 h, extracellular region (GO:0005576) was the most significantly enriched CC in FCGM-48 h. Concerning the MF, hydrolase activity (acting on glycosyl bonds; GO:0016798), hydrolase activity (hydrolysis of o-glycosyl compounds; GO:0004553), and transferase activity (transferring-glycosyl groups; GO:0016757) were the most significantly enriched in FCGM-48 h. Regarding the BP, carbohydrate metabolic process (GO:0005975), polysaccharide metabolic process (GO:0005976) and polysaccharide catabolic process (GO:0000272) were the most significantly enriched in FCGM-48 h (Fig. 2B).

#### RT-qPCR validation of RNAseq data

RT-qPCR was used to validate the 14 highly upregulated CAZyme-encoding genes. The RT-qPCR expression levels of genes were estimated as 2^−ΔΔCT^ fold change, and the trend of upregulated expression was widely consistent between the FCGM-0 h and FCGM-48 h groups (Table 3). The sequences of used primers for RT-qPCR are listed in Additional file 1: Table S4.

**Table 3:** RT-qPCR validation of selected differentially expressed genes in *B. velezensis* CL-4^1^

NCBI gene code	Enzyme description	FCGM0h ^2^ 2^−ΔΔCT^	FCGM48h^3^ 2^−ΔΔCT^
gene-K4L72_RS06255	6-phospho-β-galactosidase	4.97 ± 3.51	15.37 ± 3.78
gene-K4L72_RS04325	6-phospho-α-glucosidase	2.22 ± 1.06	881.76 ± 139.04
gene-K4L72_RS15355	α-glucosidase	1.10 ± 0.18	290.28 ± 37.98
gene-K4L72_RS01625	α-amylase	1.45 ± 0.41	4.66 ± 1.12
gene-K4L72_RS14170	Arabinan endo-1,5-alpha-L-arabinosidase	1.69 ± 0.60	3.01 ± 0.93
gene-K4L72_RS08970	xylan 1,4-beta-xylosidase	1.13 ± 0.12	21.12 ± 6.64
gene-K4L72_RS19300	Arabinan endo-1,5-alpha-L-arabinosidase	0.81 ± 0.19	354.71 ± 50.62
gene-K4L72_RS14125	α-N-arabinofuranosidase	2.89 ± 1.70	8.34 ± 1.63
gene-K4L72_RS14020	α-N-arabinofuranosidase	1.26 ± 0.23	13.56 ± 3.83
gene-K4L72_RS19275	pectin lyase	1.92 ± 1.09	94.96 ± 27.75
gene-K4L72_RS13250	acetyl xylan esterase	1.61 ± 0.54	7.29 ± 0.99
gene-K4L72_RS04250	acetyl xylan esterase	1.14 ± 0.13	40.43 ± 6.14
gene-K4L72_RS08580	acetyl xylan esterase	0.82 ± 0.19	66.54 ± 7.68
gene-K4L72_RS09050	copper-dependent lytic polysaccharide monooxygenases (LPMOs)	0.99 ± 0.15	3.63 ± 0.83

^1^ Values are means of three replicates per treatment

^2^ FCGM-0 h: CGM was inoculated with *B. velezensis* CL-4 at 37 °C for 0 h

^3^ FCGM-48 h: CGM was inoculated with *B. velezensis* CL-4 at 37 °C for 48 h

## Discussion

Jilin province has always ranked first in China for corn yield. The abundant by-products of corn wet milling have great development potential as under-utilized feed resources (Shi et al. 2019). CGM is one of the alternative protein feeds to SBM, but its high fiber content, low crude protein content, and amino acid imbalance limit its application in livestock diets (Zhang et al. 2019b). Our previous study reported that the pretreatment of CGM with sodium bicarbonate effectively solves the problem of low pH caused by corn wet milling, and improves the fermentation rate and nutritional composition of CGM (Chen et al. 2022). For instance, our process significantly improved the number of viable bacteria, enzymes (cellulase, xylanase, β-mannanase, amylase, and protease), peptides, fatty acids, and other active metabolites in CGM. Most remarkably, it increases the hemicellulose degradation rate up to 71.33%. However, whether the lignocellulose-encoding genes of *B. velezensis* exist in symbiosis and get significantly upregulated in the 48 h sample of FCGM is the key focus of this investigation.

Presently, *B. velezensis* is mostly studied for biological control and plant growth promotion effects (Wang et al. 2022). Recently, reports on the application of *B. velezensis* in livestock and poultry are gradually increasing, mainly focusing on the detoxification of mycotoxins in feed (Zearalenone and Aflatoxin B_1_) (Shu et al. 2018; Wang et al. 2021) and aquatic probiotics (Zhang et al. 2019a). However, its potential application for fermented feeds has not been explored much. *Bacillus* can enzymatically degrade plant-sourced lignocellulose (Bandounas et al. 2011). In addition, several *B. velezensis* strains such as GH1-13 (Kim et al. 2017), FZB42 (Chowdhury et al. 2015), LS69 (Liu et al. 2017), 157 (Chen et al. 2018) and LC1 (Li et al. 2020), have cellulose and hemicellulose degradation genes. Here, we performed CAZyme analysis on the twenty-three representative strains of *B. velezensis* from different sources. Many genes encoding common CAZymes were detected in the genomes of these *B. velezensis* strains*.* For instance, cellulolytic enzyme genes such as endoglucanase were common in all the examined *B. velezensis* strains. The enzymes 6-phospho-β-galactosidase, 6-phospho-β-glucosidase, 6-phospho-α-glucosidase, α-galactosidase, and β-1,3–1,4-glucanase belonging to the GH1, GH4, and GH16 families that participate in cellulose degradation, were also present in all the *B. velezensis* genomes. Furthermore, β-1,3–1,4-glucanase from the GH16 family, which has been found in other *Bacillus* species (Teng et al. 2006).

The function of CBMs is to anchor relevant enzymes to the substrate, enhance enzyme activity and increase specificity between the enzyme and substrate (Crouch et al. 2016). CBMs are present in many bacterial and fungal enzymes (Igarashi et al. 2009). CBM2 and CBM16 genes, which participate in cellulose degradation, were present in all the twenty-three *B. velezensis* strains. CBM2 is a part of many bacterial enzymes and helps enzyme binding to cellulose, xylan, and chitin (Wu et al. 2022). CBM 16 improves enzyme binding to cellulose and glucomannan (Wang and Xu 2019).

Cellulose is synergistically hydrolyzed by different enzymes, for instance, endoglucanases randomly cut the amorphous region of the cellulose polysaccharide chain to generate cellobiose(Guo et al. 2018). However, in the lack of exoculcellulase, *B. velezensis* may not directly hydrolyze cellobiose, which limits the cellulose degradation ability of *B. velezensis*. All the twenty-three *B. velezensis* strains lack the enzymes for complete cellulose degradation, and therefore it is necessary to add appropriate exogenous enzymes for collaborative treatment. In addition, common genes from the GH11, GH43, GH51, and GH30 families participate in hemicellulose degradation including endo-β-1,4-xylanase, arabinoxylan arabinofuranohydrolase, arabinan endo-1,5-α-L-arabinosidase, 1,4-β-xylosidase, α-N-arabinofuranosidase, and glucuronoxylanase and are the key factors for xylan degradation. Hemicellulose, the second most abundant lignocellulose component, can be hydrolyzed to monosaccharides by a variety of enzymatic systems (Tang et al. 2021). The catalytic site of endo-1,4-β-xylanase from the GH11 family is the β-1,4-xylosidic linkages of xylan, producing short xylooligosaccharides after xylan cleavage (Monica and Kapoor 2021). In the field of pulp bleaching, xylanases from the GH11 family are favored over xylanases from the GH10 family. The GH11 xylanases are smaller in size (~ 20 kDa), lack cellulase activity, and easily penetrate the cellulose fiber network without damaging the fiber (Bai et al. 2015). The function of β-xylosidase and β-mannosidase is to release xylose units from xylobiose and xylooligosaccharides (Barker et al. 2010), and the hydrolysis of the terminal mannose of mannan polysaccharides (Malgas et al. 2015). Some CEs (acetylxylan esterase) have the potential to deacetylate xylan and degrade xylose oligosaccharides, such as CE3, which were also found in our study. CE3 can enhance the solubilization of xylan (Zhang et al. 2011). In addition, several CE4 polysaccharide deacetylases, which participate in the degradation of plant polysaccharides, were also found in *B. velezensis* strains. In addition to the peptidoglycan N-deacetylates involved in chitin degradation, CE4 are a highly specific class of acetylxylan esterases, but they cannot degrade acetyl galactoglucomannan or acetylated manno-compounds (Biely 2012). Therefore, the hemicellulose-encoding genes that exist in *B. velezensis* may have potential applications in the food, feed, paper, and biofuel industries. Also, the PL1 (two) and PL9 (one) genes were present in all the twenty-three *B. velezensis* genomes, which participate in pectin degradation. The end of oligosaccharides is mainly subjected to elimination cleavage of (1 → 4)-α-D-galacturonan by a pectin lyase (See-Too et al. 2017). Levanase belonging to the GH32 family participates in sucrose hydrolysis (Bezzate et al. 1994). α-amylase and α-glucosidase from the GH13 family participate in starch hydrolysis (Graebin et al. 2016). There were also some auxiliary CAZymes (such as AA4, AA6, AA7, and AA10) in the twenty-three *B. velezensis* strains. Vanillyl-alcohol oxidases (VAO), a member of the AA4 family, can transform various phenolic compounds with side chains located in the aromatic ring counter-position (Xu et al. 2021). In addition, AA7 enzymes associated with biotransformation or detoxification of lignocellulosic compounds were also detected (Levasseur et al. 2013). AA10 proteins, which mainly act on chitin or cellulose, are a class of copper-dependent lytic polysaccharide monooxygenases (LPMOs) (Forsberg et al. 2014). Several *Bacillus* strains possess ligninase activity. For instance, *B. pumilus* C6 and *B. atrophaeus* B7 have laccase activity and degrade kraft lignin and dimer guaiacylglycerol-b-guaiacyl (Huang et al. 2013). *Bacillus* sp. LD003 is mainly adsorbed on the lignin component decolorizing Azure B, methylene blue, toluidine blue O and other dyes (Bandounas et al. 2011). Therefore, the presence of these lignocellulase genes in *B. velezensis* genomes suggests that the bacteria can help hemicellulose degradation, including some amount of cellulose, starch, and pectin.

Combined with the results in Table 1 and Fig. 1, in total, 108 CAZymes were found in the twenty-three *B. velezensis* strains, while the other homologous family genes showed some differences. Geographic origin and habitat can influence the function of CAZymes from lignocellulosic degrading bacteria. For example, *B. velezensis* LC1 is an endophytic bacterium isolated from the gut of *Cyrtotrachelus buqueti* that can degrade bamboo lignocellulose (Li et al. 2020). *B.velezensis* CL-4, examined in this study, is an intestinal bacterium isolated from chicken cecal contents and has non-starch polysaccharide (NSP) degradation activity. In the poultry cecum, most contents are undigested starch and NSP that are fermented by microorganisms or residual digestive enzymes to complete the digestion process (Chen et al. 2022). Some *B. velezensis* strains, such as GH1-13 (Kim et al. 2017), FZB42 (Chowdhury et al. 2015) and LS69 (Liu et al. 2017), are symbiotic rhizobacteria that can hydrolyze a series of polysaccharides, proteins and other compounds, which help their colonization in the rhizosphere. In general, the presence of such homologous family genes implies the same function and degradation mode, but the bacteria habitat may affect the specificity during the evolution of *B. velezensis*.

We performed transcriptomic analysis to compare the DEGs involved in lignocellulosic degradation that were significantly upregulated between the FCGM-0 h and FCGM-48 h samples. As shown in Table 2, GH13 is the main gene family involved in starch hydrolysis. GH43, GH51, and CE4 gene families are involved in hemicellulose hydrolysis. GH1, GH4, and PL1 gene families are involved in cellulose and pectin degradation. Notably, many highly expressed DEGs in FCGM-48 h were related to xylan degradation. The expression of xylan 1,4-β-xylosidase was found to be upregulated 5.29-folds. This enzyme attacks the β (→4 glycosidic bond on D-xylan, removing the D-xylose residue at the non-reducing end (Jamaldheen et al. 2019). The enzymes involved in xylan side chain removal, (two genes encoding α-N-arabinofuranosidase) were upregulated by 2.03 times and 3.36 times, respectively. These enzymes hydrolyze the terminal non-reducing α-L-arabinofuranoside residues and produce α-L-arabinosides (Lagaert et al. 2014). Acetoxylan esterase, an accessory enzyme, works with other enzymes to remove the backbone side-chain residues of xylan (Zhang et al. 2011). Three genes encoding acetoxylan esterases were highly expressed in this study. Genes encoding accessory enzymes involved in arabinan polymers breakdown (two genes encoding arabinan endo-1,5-α-L-arabinosidase) were upregulated by 2.15 times and 4.58 times, respectively. These enzymes improve the endo-hydrolysis of (1 → 5)-α-arabinofuranosidic linkages in (1 → 5) arabinans (Sunna and Antranikian 1997). Hence, the transcriptome analysis indicated a higher hemicellulose degradation rate in FCGM-48 h samples.

The DEGs involved in cellulose degradation mainly including maltose-6'-phosphate glucosidase and 6-phospho-β-galactosidase. The expression level of maltose-6'-phosphate glucosidase increased by 6.61 times. This enzyme hydrolyses a variety of 6-phospho-α-D-glucosides (Thompson et al. 1995). α-glucosidase and α-amylase genes, involved in starch degradation, were also upregulated by 2.13 times and 1.94 times. α-amylase participates in endo-hydrolysis of (1 → 4)-α-D-glucosidic linkages in starch, glycogen, and related polysaccharides and oligosaccharides (Janeček et al. 2014). The main role of α-glucosidase is to hydrolyze terminal non-reducing (1 → 4) -linked α-D-glucose residues and release D-glucose (Graebin et al. 2016). Pectin degradation is mainly carried out by the PL1 family of enzymes, which break down the pectin component in CGM.

The functions of GT enzymes are mainly associated with cell structure, storage, and signaling. They can biosynthesize an infinite number of oligosaccharides, polysaccharides, and glycoconjugates by transferring sugar residues from activated sugar donors to specific acceptor molecules forming glycosidic bonds (Coutinho et al. 2003). In this study, glycosyl transferases from the GT1, GT2, GT4, and GT28 families were significantly upregulated, which directly affected the biosynthesis of oligosaccharides, polysaccharides, cell wall chitin, and peptidoglycan required for bacterial metabolism (Klutts et al. 2006). Such observations of up-regulation were also found in some fungi such as *Aspergillus fumigatus* and *Aspergillus tamarii* (Miao et al. 2015). In addition, bacterial and fungal-secreted enzymes can be affected by the culture formats (Wang et al. 2010a). For *A. niger*, pretreated sugarcane bagasse improved the secretion of endoglucanase and xylanase in solid-state fermentation, while the submerged fermentation favored the production of β-glucosidase (Midorikawa et al. 2018). Therefore, culture format may also affect the *B. velezensis* secretion of lignocellulase during the fermentation of CGM. However, the enzyme production of *B. velezensis* during submerged fermentation of CGM still needs to be verified by subsequent experiments.

In conclusion, we found that *B. velezensis* CL-4 fermentation promoted arabinoxylan degradation in CGM, followed by the partial degradation of cellulose, pectin, and starch. Furthermore, transcriptome and CAZyme analysis revealed the common lignocellulase in the twenty-three strains of *B. velezensis*, which can be utilized for the degradation of cellulose and hemicellulose components of biomass. The different characteristics of *B. velezensis* secreted lignocellulase may be related to their habitats. Importantly, exogenous cellulase can be combined with *B. velezensis* to further improve the degradation of cellulose components in CGM.

## Supplementary Information

**Additional file 1**: ** Figure S1**. Principal components analysis (PCA) of FCGM-0h (A) and FCGM-48h (C) samples. **Figure S2**. Correlation test of FCGM-0h (A) and FCGM-48h (C) samples. **Table S1**. Comparative genomic analysis of CAZymes in the twenty-three *B. velezensis* strains. **Table S2**. The quality of transcriptomic sequencing data for FCGM-0h and FCGM-48h samples. **Table S3**. The result of transcriptomic sequencing data compared with the reference genome of *B. velezensis* CL-4. **Table S4**. Primer sequences used for the RT-qPCR validation of seq data (14 DEGs).

## Data Availability

All data analyzed during this study are included in this manuscript and additional material.
