# Supplementary material for: Comparative genomic and transcriptome analysis of Bacillus velezensis CL-4 fermented corn germ meal
Source: AMB Express. 2023 Jan 23;13:10. doi: 10.1186/s13568-023-01510-5 (PMC9868226; doi:10.1186/s13568-023-01510-5)
Supplement: Supplementary file 1 — Additional file 1: Figure S1. Principal components analysis (PCA) of FCGM-0h (A) and FCGM-48h (C) samples. Figure S2. Correlation test of FCGM-0h (A) and FCGM-48h (C) samples. Table S1. Comparative genomic analysis of CAZymes in the twenty-three B. velezensis strains. Table S2. The quality of transcriptomic sequencing data for FCGM-0h and FCGM-48h samples. Table S3. The result of transcriptomic sequencing data compared with the reference genome of B. velezensis CL-4. Table S4. Primer sequences used for the RT-qPCR validation of seq data (14 DEGs). [file 13568_2023_1510_MOESM1_ESM.pdf]

AMB express

Comparative genomic and transcriptome analysis of *Bacillus velezensis* CL-4 fermented corn germ meal

Long Chen[1], Zihui Qu[1], Wei Yu[1], Lin Zheng[1], Haixin Qiao[3], Dan Wang[1], Bingdong Wei[1*], Zijian Zhao[2*]

Institutions:

[1]Institute of Animal Nutrition and Feed, Jilin Academy of Agricultural Sciences, Jilin Gongzhuling 136100, China;

[2]Institute of Agro-food Technology, Jilin Academy of Agricultural Sciences, Changchun, 130033, China;

[3] Information Application Department, Jilin Intellectual Property Protection Center, Changchun, 130000, China.

Corresponding authors:

Bingdong Wei

Institute of Animal Nutrition and Feed, Jilin Academy of Agricultural Sciences, No. 186 Dong Xinghua Street, Gongzhuling, Jilin Province, 136100, P.R. China

Tel: +86-0434-5163761; Fax: +86-0434-5163761; Email: weibingdong@dlut.edu.cn

Zijian Zhao

Institute of Agro-food Technology, Jilin Academy of Agricultural Sciences, No. 1366 Cai Yu Street, Changchun, Jilin Province, 130033, P.R. China

Tel: +86-0431-87063285; Fax: +86-0431-87063285; Email: zhaojaas@163.com.

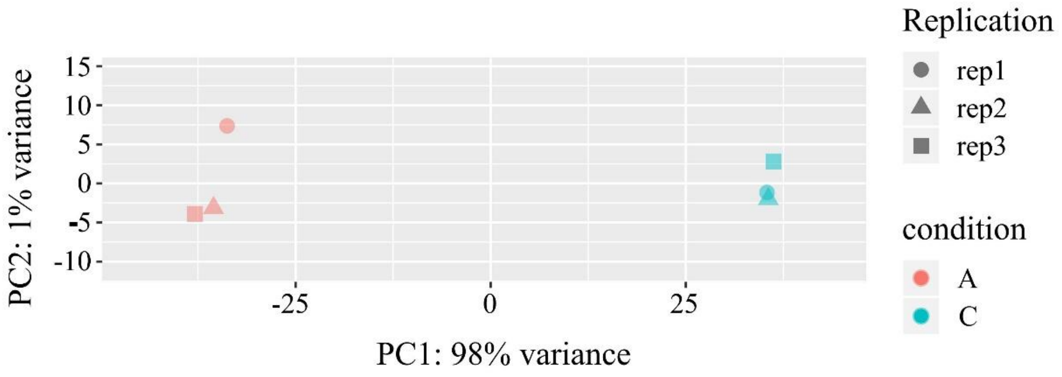

Fig. S1. Principal components analysis (PCA) of FCGM-0h (A) and FCGM-48h (C) samples.

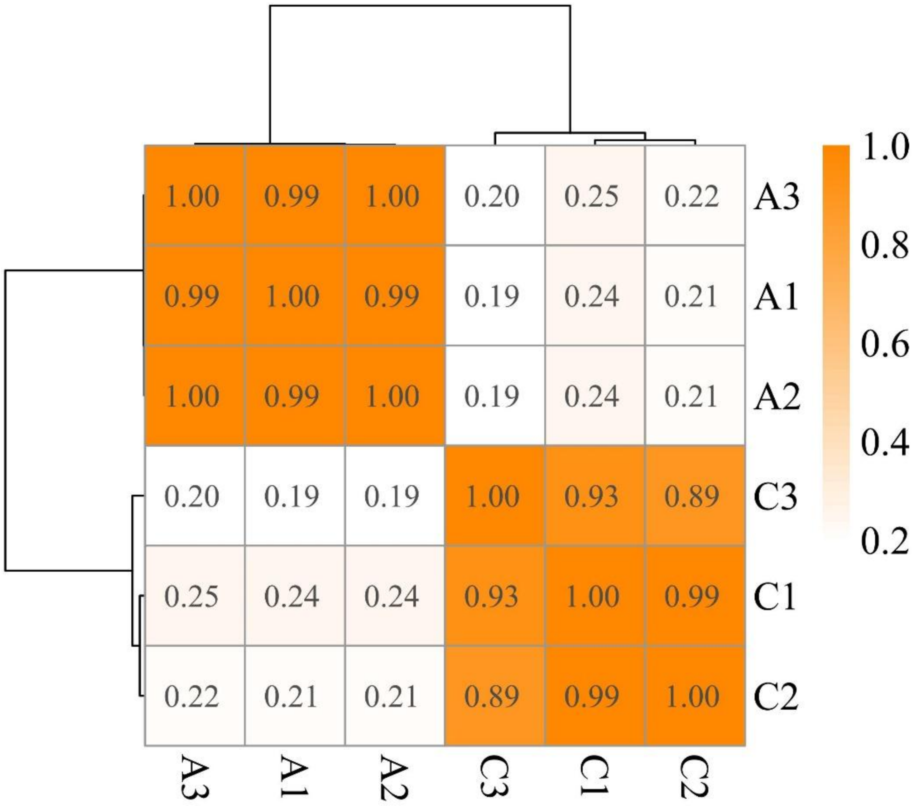

Fig. S2. Correlation test of FCGM-0h (A) and FCGM-48h (C) samples.

Table S1. Comparative genomic analysis of CAZymes in the twenty-three *B. velezensis* strains

B.velezensis	Source	Size（Mb）	GC%	Biosample	Bioproject	GH	GT	PL	CE	AA	CBM
LC1	*Cyrtotrachelus buqueti*	3.93	46.50	SAMN12838279	PRJNA574012	45	38	3	30	7	15
S3-1	Rhizosphere soil of cucumber	3.93	46.50	SAMN04816858	PRJNA318322	45	38	3	30	7	14
LS69	Rice field	3.92	46.50	SAMN05172932	PRJNA322716	45	38	3	30	7	14
83	Mango tree phyllosphere	4.00	46.40	SAMN10268423	PRJNA497887	48	38	3	29	7	14
FZB42	Infected sugar beet	3.92	46.50	SAMN02603158	PRJNA13403	44	38	3	31	7	13
LPL-K103	Surface of a lemon	3.93	46.60	SAMN03272507	PRJNA271043	43	33	3	31	5	18
157	Bark of *Eucommia ulmoides*	4.02	46.39	SAMN07311555	PRJNA392845	48	39	3	29	7	15
JTYP2	Leaves of *Echeveria laui*	3.93	46.50	SAMN06617410	PRJNA379631	44	38	3	30	7	14
UCMB5036	Cotton plant	3.91	46.60	SAMEA2272633	PRJEB1155	45	38	3	31	7	18
DR-08	Not described	3.93	46.50	SAMN08826799	PRJNA448279	45	38	3	30	7	14
B25	the inner wood tissues of a decaying Platanus X acerifolia tree	3.86	46.69	SAMEA3726545	PRJEB12378	44	38	3	32	7	15
At1	Seedlings of *Arabidopsis thaliana* germinated aseptically from surface sterilized seeds	3.89	46.70	SAMN12058370	PRJNA176703	46	38	3	29	7	18
GFP-2	from the *Chiloscyllium plagiosum* (Whitespotted bamboo shark) intestine	3.98	46.40	SAMN06670433	PRJNA381111	44	39	3	32	7	13
CL-4	Chicken cecal contents	4.06	46.30	SAMN20752450	PRJNA754316	48	38	3	27	7	16
CC09	healthy leaves of *Cinnamomum camphora*	4.17	46.10	SAMN04550342	PRJNA315173	47	38	3	31	7	16
LDO2	peanut root	3.95	46.50	SAMN08971201	PRJNA451464	45	38	3	30	7	14
9D-6	Soil samples were collected from Blizman potato fields	3.96	46.40	SAMN06711603	PRJNA382661	47	39	9	29	7	15

ANSB01E	Chicken cecal content	3.93	46.50	SAMN10963365	PRJNA523053	44	37	3	30	7	14
M75	Cotton waste	4.01	46.60	SAMN05323145	PRJNA327161	47	39	3	32	7	16
JT3-1	Domestic yak	3.93	46.50	SAMN10080444	PRJNA491562	45	38	3	30	7	14
A2	Not described	3.93	46.50	SAMN14912738	PRJNA632588	45	38	3	30	7	14
NJN-6	isolated from the rhizosphere soil of healthy banana plants	4.05	46.60	SAMN03492032	PRJNA236411	48	39	3	32	7	15
LF01	*Nile tilapia* (Oreochromis niloticus)	3.97	46.60	SAMN15336677	PRJNA640882	45	38	3	32	7	18

Table S2. The quality of transcriptomic sequencing data for FCGM-0h and FCGM-48h samples.

Sample	Total number of Reads	total number of bases	Q30 (bp)[1]	N (%)[2]	Q20 (%)[3]	Q30 (%)[4]
FCGM0h-1	28173650	4226047500	3862914107	0.05	96.80	91.40
FCGM0h-2	29059462	4358919300	4066036267	0.05	97.49	93.28
FCGM0h-3	29908216	4486232400	4199309257	0.05	97.65	93.60
FCGM48h-1	31480952	4722142800	4387841593	0.05	97.35	92.92
FCGM48h-2	28772014	4315802100	4014778802	0.05	97.39	93.02
FCGM48h-3	29958328	4493749200	4208753846	0.05	97.66	93.65

[1]Q30 (bp): The total number of bases with recognition accuracy < 99.9%；

[2]N(%): The percentage of fuzzy bases；

[3]Q20(%): The percentage of bases with base recognition accuracy < 99%；

[4]Q30(%): The percentage of bases with base recognition accuracy < 99.9%.

Table S3. The result of transcriptomic sequencing data compared with the reference genome of *B. velezensis* CL-4.

Sample	Useful Reads[1]	Total Mapped Reads[2]	(%)	Uniquely Mapped Reads[3]	(%)	Multiple Mapped Reads[4]	(%)
FCGM0h-1	26490254	26074804	98.43	25691882	98.53	382922	1.47
FCGM0h-2	26844936	26451830	98.54	26110249	98.71	341581	1.29
FCGM0h-3	28153930	27716651	98.45	27384036	98.80	332615	1.20
FCGM48h-1	29821718	29318313	98.31	28599426	97.55	718887	2.45
FCGM48h-2	27272892	26633585	97.66	25790954	96.84	842631	3.16
FCGM48h-3	27359786	26942971	98.48	26299214	97.61	643757	2.39

[1]Useful Reads: The total number of sequences used for alignment.
[2]Total Mapped Reads (%): The total number and proportion of sequences in the reference genome.
[3]Uniquely Mapped Reads (%): The total number and proportion of sequences aligned to a single position.
[4]Multiple Mapped Reads (%): The total number and proportion of sequences aligned to multiple locations.

Table S4. Primer sequences used for the RT-qPCR validation of seq data (14 DEGs).

Primer name	forward sequence (5'to3')	Reverse sequence（5'to3'）
K4L72_RS01625	TGCAAACGCTGAAACTGCAA	TAGCCCGCATCACGAATCTC
K4L72_RS04250	ACCCCGAAAGTGCTTGATGT	TGCCGATGATATGGCCTTCC
K4L72_RS04325	TGGAAACGTACTCGCCTGAC	TTCGCCATGCGATCCTCAAT
K4L72_RS06255	CAAACGGCGCTTCATACGAG	TATCGCCCGTTCCGTTATGG
K4L72_RS08580	TGAAGCCGAAATGGTTTGCG	TGTAAAACAGCGGGAGACGG
K4L72_RS08970	TAACCGCAGAAGGAGGAACG	TGGGTTTCCTGGATCATGCC
K4L72_RS09050	CAAATGGGGGATCGGGACAA	GTCGCATGAGGAGCTGTGTA
K4L72_RS13250	TGGAGGGCTTGGTGACTTTC	GGATTCGCAGTCAAACCTGC
K4L72_RS14020	GCGGCTGTTTTGCTGATGAA	TTACCGGCGATGTACGGTTC
K4L72_RS14125	CAGAACAGACGTGCAGGAGT	CATGCCAAATCAAGCCTCCG
K4L72_RS14170	AAAGACGGAAACCCTTGGCT	CAAAGCTCCGCCGTTATTCG
K4L72_RS15355	TTGGGAAAGCATTTTCGGCG	CCACCAGCGAACCATCTCAT
K4L72_RS19275	CATCTCGCTTTCTTGGCTGC	ACGGCGGATTTTGCTTTTCC
K4L72_RS19300	TTTCAGAGGTCAGCGTGCAT	GCTTGAAGACAGTTGCTGCC
16s	ACTCCTACGGGAGGCAGCA	GGACTACTGGGGTTTCTAAT
